# Making the Needed Linkages and Economic Case for Continued Lead-Paint Abatement

**DOI:** 10.1289/ehp.13098

**Published:** 2009-08

**Authors:** Andrew M. Geller

**Affiliations:** National Health and Environmental Effects Research Laboratory, Office of Research and Development, U.S. Environmental Protection Agency, Research Triangle Park, North Carolina, E-mail: geller.andrew@epa.gov

The connection between environmental public health regulations, regulatory science, and public health gains can, at times, be difficult to elucidate. Why? Some challenges are raised by the complex mosaic of federal and state regulatory decisions, differences in the implementation of those decisions at more local levels, the multiple determinants of disease, and temporal lags between exposure and ultimate health outcomes (National Research Council 2007). This difficulty in making linkages between regulations and health outcomes often necessitates the choice of indirect measurements of success such as process indicators (e.g., number of facilities inspected) or emissions indicators (e.g., tonnage of pollutants captured). Such indirect indicators fail to make the necessary connection to human exposure or health outcomes. The challenge remains to make these linkages at the appropriate spatial and temporal scales to bolster the evidence base for environmental decision-making [U.S. Environmental Protection Agency (EPA) 2008b].

Despite these difficulties, it remains incumbent on the environmental regulatory community to, where possible, “close the loop” by demonstrating that regulations have achieved their intent. Developing ways to better understand the impact of regulations and policies is essential, and in the long run will help regulators to predict the public health impacts of incremental or major changes in regulatory actions that affect environmental exposures (U.S. EPA 2007). This is a critical step in ensuring that the actions taken and resources spent by governments, the regulated community, and others are protecting public health. Further, it helps to identify where continued improvements could be made.

One notable area where the linkage between regulation and health outcomes has been made is in lead abatement. Figure 1 provides a concise view of how actions promulgated to reduce lead exposure can be arrayed across a hierarchy of indicators, making the necessary connections between rules, actions, and outcomes that are necessary to evaluate results and plan next steps. Gaps in this chain that still existed in the 1970s, such as the relationship between air lead and blood lead, presented considerable obstacles to the promulgation of the health-based regulation limiting lead content of gasoline (Bridbord and Hanson 2009). That these linkages can now be made is testament to both the magnitude and ubiquity of the problem of lead contamination, the broad engagement of the scientific community over decades of research, and excellent planning to evaluate the outcomes of lead mitigation (e.g., Galke et al. 2005). Bridbord and Hanson’s (2009) narrative makes the important point that taking the lead out of gasoline has made possible further examination of the health effects of lower and lower levels of lead contamination, an example of where following up on the outcomes of regulatory activities has identified opportunities for further risk mitigation and public health promotion.

In the July 2009 issue of *Environmental Health Perspectives*, Gould (2009) used data accumulated over the past decade on the linkage between environmental lead exposures and health effects to present a compelling description of the potential costs and benefits of lead hazard control, concentrating on the residential lead paint hazard. In her analysis, Gould incorporated certain health, social, and behavioral costs and benefits left out of earlier valuation analyses associated with lead (President’s Task Force 2000; U.S. EPA 2008a, 2008c) arguing, in effect, that these data have reached a level of maturity that permits their inclusion.

Gould had good reason for concentrating on lead paint for her analysis; 25% of the nation’s housing has significant hazards from lead-based paint in the form of deteriorated paint, dust lead, or bare soil lead (Jacobs et al. 2002). Abatement is a good indicator of success. The accumulated evidence is clear: Abatement reduces dust-lead loadings when measured as far out as 3 years after treatment (Dixon et al. 2005). This abatement results in decreases in whole blood lead levels of approximately 20–26% within a year for population samples of children with preabatement levels just above the current intervention level (geometric mean, 11 μg/dL; Galke et al. 2001) and at higher levels (23 μg/dL; Taha et al. 1999).

Moreover, some analyses (e.g., Nriagu et al. 2006) suggest that the remaining problem of lead contamination is likely associated with the presence of peeling paint and cracked walls in housing rather than with variables associated with water use or potential air exposure. This applies even in an urban system with lead present in the water distribution system or with proximity to possible outdoor sources of lead contamination such as factories, incinerators, repair shops, or gas stations.

Gould’s focus on paint hazard (Gould 2009) highlights the dramatic decrease in the level of lead exposure and associated biomarker levels due to the reduction of lead content in gasoline, household paint, industrial emissions, drinking water, food canning, and ceramic glazes. It also acknowledges, however, that a large problem remains. In Milwaukee, Wisconsin, for example, 15.7% of children living in neighborhoods surrounding a former industrial corridor have blood lead levels ≥ 10 μg/dL [Agency for Toxic Substances and Disease Registry (ATSDR) 2008]. These children and others in lead exposure hot spots have not fully shared the benefits of the regulatory and mitigation actions that have brought the national average down to 1.5 μg/dL (U.S. EPA 2008b). The magnitude of this problem remains a public health challenge, and the associated lead exposure hazard is an obstacle to the revitalization of America’s cities and communities.

U.S. EPA Administrator Lisa Jackson has commented on a number of occasions that science must be the determining factor in making decisions that affect the environmental health of America’s communities, that the determinations and process of our decisions must be transparent, and that eliminating disparities in the environmental health of America’s communities must be paramount (Jackson 2009a, 2009b, 2009c). The analysis by Gould (2009) offers an opportunity in all of these areas by bringing a broad consideration of publicly available medical, economic, and social sciences to bear on the well-developed understanding of the sources and routes of lead exposure. Further, Gould identifies and quantifies the costs and benefits of closing the book on a major remaining facet of our nation’s lead problem. In a real sense, she offers lead abatement as an example of a “green economy” for which modest investment can reap great returns. She also points to the importance, at this time, of considering the nation’s environmental health issues in a place-based context, showing that while dramatic, initial environmental health gains have been made based on sector-based banning of lead from consumer products and through air and water regulation, the public health and economically critical increments that remain will need to be addressed through place-based targeting for a burden that disproportionately affects residents with lower socioeconomic status.

Even with the inclusion of these additional factors, Gould’s estimate (Gould 2009) might still be considered conservative because it concentrates almost wholly on children < 6 years of age. Recent research has identified associations between cumulative lead exposure and cognitive impairment, hypertension, and other health outcomes in older adults (Hu et al. 2007; Navas-Acien et al. 2007; Shih et al. 2007). These adverse outcomes are worsened by coexposure to stress, including neighborhood psycho social hazards (Glass et al. 2009; Peters et al 2007; cf. Gump et al. 2008). Although much of the research on older adults has focused on lead accumulated and stored in bone during the lifetime, questions still remain about the role of concurrent exposure to lead from housing stock or other sources (Lin et al. 2004).

Most older Americans age in place, with 29% of adults > 65 years of age living in housing stock built in 1949 or earlier. This is especially true for urban and rural communities in the Northeast and Midwest regions of the United States, where 40–60% of older adults live in this housing stock (Golant 2008). Unfortunately, these are also the regions where lead-based paint hazards are most prevalent, particularly in this older housing; 57% of housing built in 1940–1959 and 81% built before 1940 contain lead hazards (Jacobs et al. 2002), and risk of lead toxicity for children is highest in these regions, particularly in older housing stock (Bernard and McGeehin 2003). If the relationship between older housing stock and the percentage of older adults with elevated blood lead remotely mirrors that in children (Bernard and McGeehin 2003; Sargent et al. 1997), then the problem of concurrent exposure may indeed be a serious one.

In her analysis, Gould (2009) demonstrated significant benefits to improving public health by lead paint remediation or control, adding an economic basis to the health and exposure data that make a process indicator such as paint hazard abatement a reasonable surrogate for exposure and health indicators for children. If the linkages found for children also apply to older adults, it will be important to assess whether additional, economically quantifiable benefit can be gained from remediation aimed at aging adults, the most rapidly growing portion of the U.S. population.

## Figures and Tables

**Figure 1:**
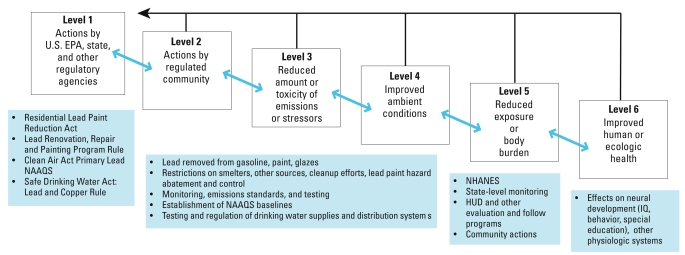
The hierarchy of indicators described by the U.S. EPA (2007) and some examples of the indicators at these different levels related to environmental lead contamination. Abbreviations: HUD, U.S. Department of Housing and Urban Development; NAAQS, National Ambient Air Quality Standard; NHANES, National Health and Nutrition Examination Survey. Linkages made between these indicators can be used to evaluate the efficacy of current actions and to predict the potential impact of proposed actions. For a more detailed account of federal roles in lead paint abatement, see the President’s Task Force report (2000).

**Figure:**
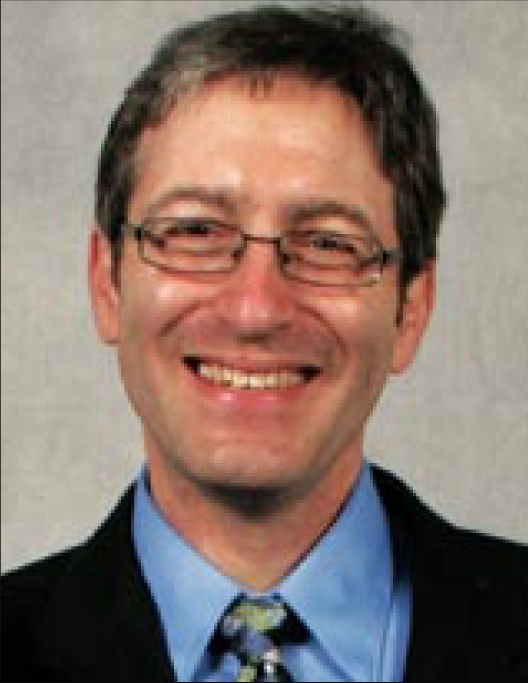
Andrew M. Geller
